# Prevalence of bacterial infections and factors associated with death related to these infections in two medical departments of a tertiary hospital in Dakar, Senegal

**DOI:** 10.1016/j.ijregi.2025.100623

**Published:** 2025-03-08

**Authors:** Moustapha Diop, Chancia Guitoula, Ajuamendem Ghogomu Tamouh, Tracie Youbong, Sokhna Moumy Mbacké Daffé, Maguette Ndoye, Mamadou Wagué Gueye, Fatimata Wone, Mor Ngom, Mamadou Seck, Nogaye Youm, Oumar Bassoum, Ndèye Aissatou Lakhe, Papa Samba Ba, Adama Faye, Sarra Boury Gning

**Affiliations:** 1 Department of Infectious and Tropical Diseases, Dakar Principal Hospital, Dakar, Senegal; 2 Private Institute of Medical Training and Research, Dakar, Senegal; 3 Department of Human Resources, Ministry of Public Health, Yaoundé, Cameroon; 4 Laboratories Federation, Dakar Principal Hospital, Dakar, Senegal; 5 Department of Infectious and Tropical Diseases, Dalal Jamm Hospital, Dakar, Senegal; 6 Department of Infectious and Tropical Diseases, FANN Teaching Hospital, Dakar, Senegal; 7 Health and Development Institute, Cheikh Anta DIOP University, Dakar, Senegal; 8 Internal Medicine, Dakar Principal Hospital, Dakar, Senegal

**Keywords:** Associated factors, Bacterial infections, Dakar, Death, Multi-drug-resistant bacteria

## Abstract

**Objectives:**

This study aimed to estimate the prevalence of bacterial infections and identify the factors associated with death related to these infections in the internal medicine (Brévié) and infectious diseases departments (Boufflers) of Dakar Principal Hospital.

**Methods:**

This cross-sectional study was carried out from January 1 to December 31, 2023, including patients with bacterial infections hospitalized in Boufflers and Brévié. Multivariate logistic regression was used to identify factors associated with death.

**Results:**

Out of 1,085 hospitalized patients, 181 (16.7%) had bacterial infections. The mean age was 60±18 years, with a sex ratio of 1.08. Urinary tract infection (37%) was the most represented clinical presentation. Bacteria were isolated in 123 patients (68%), with *Escherichia coli* (39%), *Staphylococcus aureus* (12%), and *Klebsiella pneumoniae* (12%) as the most represented species. Of the 140 identified bacteria, 78 (55.7%) were multidrug-resistant. The death rate was 15%. Multivariate analysis showed that age ≥ 65 years (odds ratio [OR] = 3.2; 95% confidence interval [CI] 1.2-9.5), prior hospitalization (OR = 2.9; 95% CI 1.1-8.5), and hemoglobin levels between 3.4 g/dl and 7 g/dl (OR = 11.7; 95% CI 2.5-60) or between 8 g/dl and 11 g/dl (OR = 4.9; 95% CI 1.6-18.4) compared with levels ≥11 g/dl were associated with death.

**Conclusions:**

This study showed a high prevalence and mortality rate of bacterial infections in the internal medicine and infectious diseases departments, particularly among older adult patients, those with a history of hospitalization, or those with low hemoglobin levels.

## Highlights

• The prevalence of bacterial infection is high in the internal medicine and infectious diseases departments of Dakar Principal Hospital.
• The top three bacterial species isolated were *Escherichia coli, Staphylococcus aureus*, and *Klebsiella pneumoniae*.
• More than half of the isolated bacteria were multidrug-resistant.
• Bacterial infections represent a major cause of death in the medical departments of Dakar Principal Hospital.
• Patients with advanced age, prior hospitalization in the previous 6 months, and a low rate of hemoglobin were more at risk of dying.

## Introduction

Bacterial infections are a major health problem, notably in low- and middle-income countries. According to a global review published in 2022, of the 13.7 million infection-related deaths in 2019, 7.7 million were associated with 33 bacterial agents, representing 13.6% of all global deaths [1]. The age-standardized mortality rate associated with these bacterial agents was the highest in sub-Saharan African countries, with 230 deaths per 100,000 inhabitants [1]. More than 6 million deaths occurred due to at least one of these three clinical presentations: lower respiratory tract infections, bacteremia, and intra-abdominal infections [1]. This heavy burden in the world is exacerbated by the inception of multidrug-resistant (MDR) bacteria. According to the 2021 report of the Global Antimicrobial Resistance Surveillance System (GLASS), 36.6% of *Escherichia coli* strains isolated from blood cultures were resistant to ceftriaxone and 24.9% of *Staphylococcus aureus* strains were resistant to methicillin. The overall resistance rates to ceftriaxone for *Klebsiella pneumoniae* and *E. coli* strains isolated from urine samples varied between 40% and 50%, and the resistance to carbapenems for *Acinetobacter* spp isolated from blood samples was 65.5% [2]. The number of deaths attributed to bacterial antibiotic resistance was estimated at 1.27 million in 2019. The majority of these deaths were reported in sub-Saharan and Western Africa, with 27.3 deaths per 100,000 inhabitants [3]. In Senegal, according to a study conducted in Saint Louis, out of 272 strains of enterobacteria isolated from urine samples, 102 (37.5%) were broad-spectrum beta-lactamase producers, mainly *E. coli* (52%) and *Klebsiella* spp (40%) [4]. A study on health care-related infections at the FANN, National University Teaching Hospital (Dakar) showed that 65.5% (16/26) of *S. aureus* strains isolated were methicillin-resistant, whereas 28.6% (2/7) and 70% (7/10) of *Pseudomonas* and *Acinetobacter* strains were resistant to ceftazidime, respectively [5]. Similarly, carbapenem-resistant strains of *Acinetobacter baumannii* and enterobacteria have also been described in Dakar [6,7]. Most of the studies on infectious diseases focus on specific pathogens such as *Mycobacterium tuberculosis, Plasmodium* spp, HIV, or hepatitis virus. Few studies have focused on bacterial infections. They are often limited to a small number of infectious sites, specific populations such as patients living with HIV and children, or certain bacteria like *Streptococcus pneumoniae* and *Neisseria meningitidis* in the case of bacterial meningitis. This is why we conducted this study with the goal of estimating the prevalence of bacterial infections and identifying the factors associated with death related to these infections in the internal medicine and infectious diseases departments of the Principal Hospital of Dakar (DPH).

## Patients and methods

### Study period and design

We conducted a cross-sectional study over a 1-year period from January 1, 2023 to December 31, 2023. Our study population was patients admitted to the internal medicine and infectious diseases departments of the DPH during the study period. Among them, patients diagnosed with a bacterial infection based either on microbiologic documentation or epidemiological, clinical, and/or paraclinical arguments were eligible for the study. Patients with either specific bacterial infections (tuberculosis or other mycobacteria) or unexploitable medical files were not included. Bacteriologic identification was done after direct examination using automated techniques (Vitek2) or classic techniques (Api gallery). The resistant profiles were described using an antibiogram according to the 2023 guidelines of the Antibiogram Committee of the French Society of Microbiology (CASFM2023).

### Data collection

Data were collected from the medical records by a trained investigator. The sampling method was exhaustive, taking into account all hospitalized patients with bacterial infections. A previously tested case report form was used to collect information from each participating patient. The data collected included sociodemographic characteristics (age, gender, marital status, and address lifestyle), comorbidities such as diabetes, high blood pressure, HIV infection, chronic kidney disease, and other health conditions, hospitalization over the past 6 months with the duration and if there was an invasive device, previous antibiotic use in the past 6 months, clinical features, the site of infection, presence of invasive devices during the current hospitalization, microbiologic data, the type of infection (nosocomial or community-acquired), antibiotics received, and patient outcome.

### Definition of terms

The following definitions were considered during the analyses:

- Sepsis was defined as the presence of a clinically suspected or microbiologically documented bacterial infection associated with at least two of the following quick Sequential Organ Failure Assessment signs: a Glasgow score <13, systolic blood pressure ≤100 mmHg, or respiratory rate ≥22 breaths per minute.
- A bacterium was considered MDR if it resisted antibiotics from at least three different classes [8].
- An infection was classified as nosocomial if it either occurred on the third day following admission or on the first or second day following hospitalization if the patient came from another hospital within 48 hours before the new admission.
- Positive C-reactive protein (CRP) = CRP ≥ 6 mg/l.

### Data management and statistical analysis

Data were recorded in the Kobocollect electronic questionnaire, exported to Excel software, and analyzed using R software (version 4.3.3). According to the distribution, quantitative variables were represented using either means and SD or medians and their interquartile ranges. Qualitative variables were represented using frequency and percentages. The binary variable “death occurrence” was considered the variable of interest with “Yes” or “No” responses. The Student's *t*-test or Wilcoxon-Mann-Whitney test, depending on their applicability, was used to compare the means. To compare the proportions of potentially explanatory qualitative variables, we used Pearson's chi-square test, Yates’ corrected chi-square test, or Fisher's test, depending on their applicability. After checking the distribution normality and the homogeneity of variances, the means of potentially explanatory quantitative variables were compared using either the Student's *t*-test or Wilcoxon-Mann-Whitney test. The potentially explanatory qualitative variables were compared using the Pearson chi-squared test, chi-squared test with Yates' continuity correction, or Fisher's exact test.

To identify the factors associated with death, we performed a univariate analysis and then a multivariate logistic regression to calculate the adjusted odds ratios (aOR) and their respective 95% confidence intervals (CIs). All independent variables with a *P*-value <0.2 in the univariate model were introduced in the multivariate model, and a backward stepwise method was used to generate the final model. Hosmer Lemeshow's adequation test and interaction checking between independent variables were performed to validate the final model with a p-value <0.05. At the end of this procedure, independent variables were significantly associated with death when the 95% CI of their aOR excluded the value 1.

### Ethical considerations

This study was conducted in accordance with the revised version of the Declaration of Helsinki and the principles of the International Conference on Harmonization Good Clinical Practice (GCP-ICH E6) after obtaining authorization from the heads of the internal medicine and infectious diseases departments. Strict confidentiality of the collected information, the anonymity of study participants was maintained and data were recorded in a secure database. An identification code was assigned to each patient, and only the initials of the names appeared on the collection tool. The risk incurred during the implementation of this survey is considered minimal, either physically, emotionally, psychologically, legally, socially, or economically. The study protocol was reviewed and approved by the ethical committee of the Idrissa Pouye General Hospital (IPGH).

## Results

**Figure 1:**
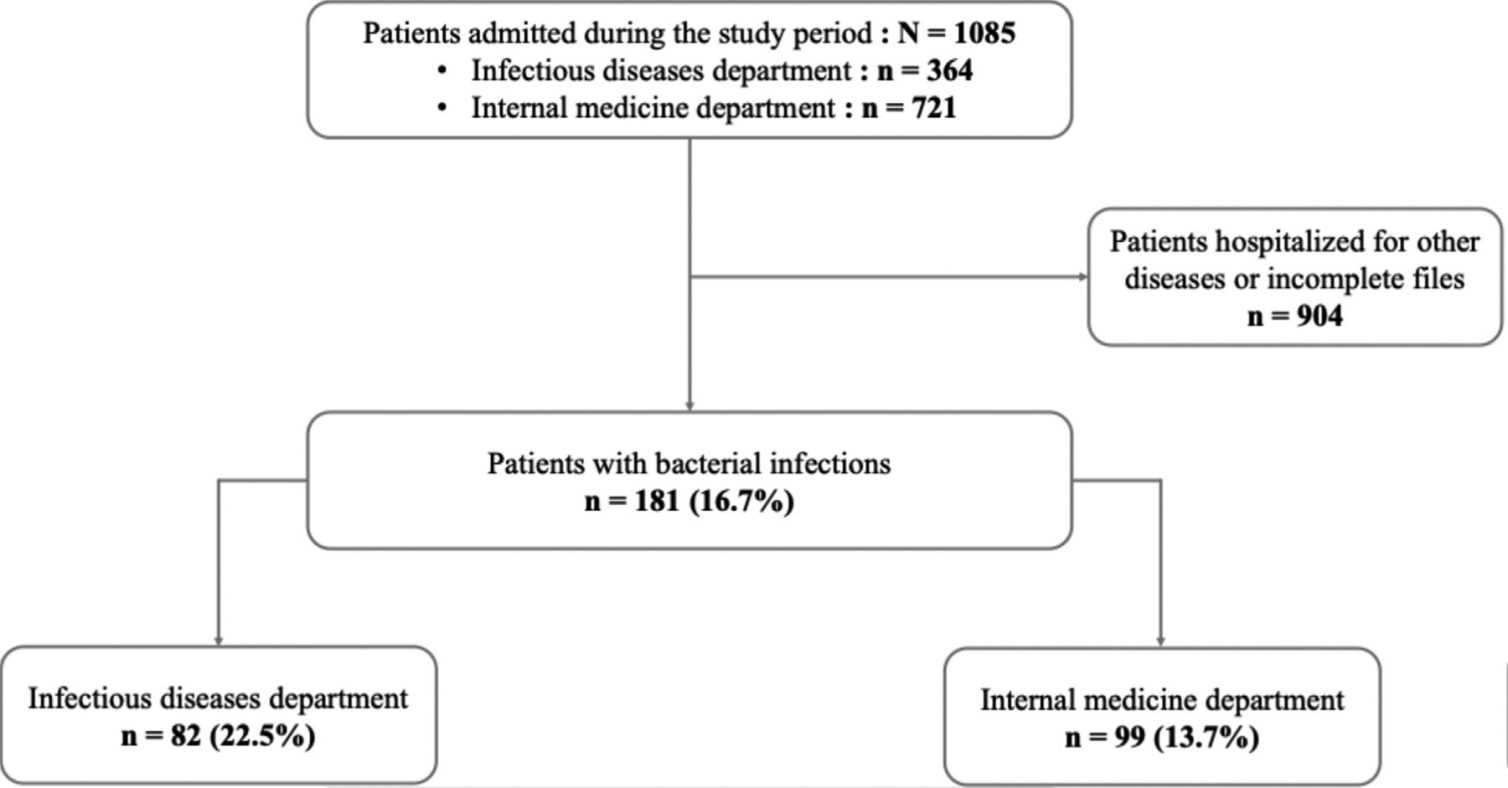
Flowchart of patients with bacterial infection in the infectious diseases and internal medicine departments of DPH from January 1, 2023 to December 31, 2023 (N = 1,085). DPH, Principal Hospital of Dakar.

Out of 1,085 hospitalized patients during the study period, 181 had a bacterial infection, representing a prevalence rate of 16.7%. (i.e., 22.5% in the infectious diseases department [82/364] and 13.7% [99/721] in the internal medicine department) (Figure 1).

### Baseline characteristics

The mean age of the study population was 60±18 years, with the majority of patients being in the 60-80 age group (38%). Males were slightly predominant, with a sex ratio of 1.1. In the 6 months preceding inclusion, 80 patients (44%) had been hospitalized at least once. Of these, 11 patients (14%) were admitted to the surgical departments, whereas 69 patients (86%) were hospitalized in the medical departments. During previous hospitalizations, 92.5% (74/80) had at least one invasive device, the most common being peripheral venous catheters (99%) and urinary catheters (34%). The median duration of prior hospitalizations was 9 days (range: 1-35 days). In addition, 56 patients (31%) had been treated with antibiotics in the 6 months prior to the study. With regard to comorbidities, diabetes (43%) and hypertension (31%) were the most frequently reported conditions (Table 1).

**Table 1:** Baseline characteristics of patients with non-tuberculous bacterial infections in the infectious diseases and internal medicine departments of DPH from January 1, 2023 to December 31, 2023 (N = 181).

Characteristic	Number	Percentages (%)
**Age group (years old)**		
17-40	29	16
41-60	59	33
61-80	69	38
>80	24	13
**Gender**		
Female	86	48
Male	95	52
**Marital status**		
Single	16	8.8
Divorced	3	1.7
Married	127	70
Widowed	35	19
**Smoking**		
Non-smokers	157	87
Current smokers	9	5
Past smokers	15	8
**Previous hospitalization in the last 6 months**	80	44
**Presence of invasive devices**	74	93
**Previous antibiotic therapy in the last 6 months**	56	31
**High blood pressure**	75	41
**Diabetes**	78	43
**Chronic kidney disease**	10	5.5
**Heart disease**	8	4.4
**Asthma**	7	3.9
**Autoimmune disease**	9	5
**Stroke**	7	3.9
**Cancer**	6	3.3
**HBV**	5	2.8
**HIV**	11	6.1

DPH, Principal Hospital of Dakar; HBV, Hepatitis B Virus.

### Clinical features

**Figure 2:**
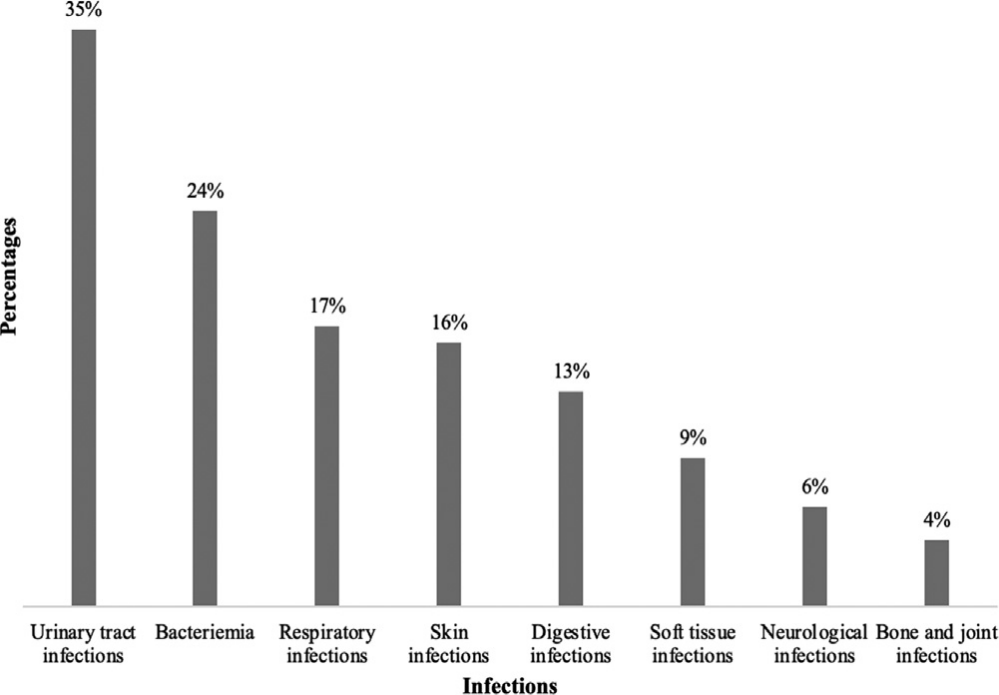
Repartition of patients with bacterial infections as per the site of infection in the Infectious Diseases and Internal Medicine departments of DPH from January 1, 2023 to December 31, 2023 (N = 181). DPH, Principal Hospital of Dakar.

In our study, 70% of patients (127/181) had community-acquired infections, 27% (49/181) had health care-associated infections, and 3% (5/181) presented with both community-acquired and health care-associated infections. The mean body temperature of the study population was 37.1ºC±1ºC, with 20% (36/181) being febrile. The quick Sequential Organ Failure Assessment assessment indicated that 4.4% (8/181) of patients had sepsis. Urinary tract infections were the most common clinical presentation, with 61 cases (35%), followed by bloodstream infections and respiratory infections, accounting for 24% (44/181) and 17% (30/181) of cases, respectively (Figure 2).

### Bacteriology

**Figure 3:**
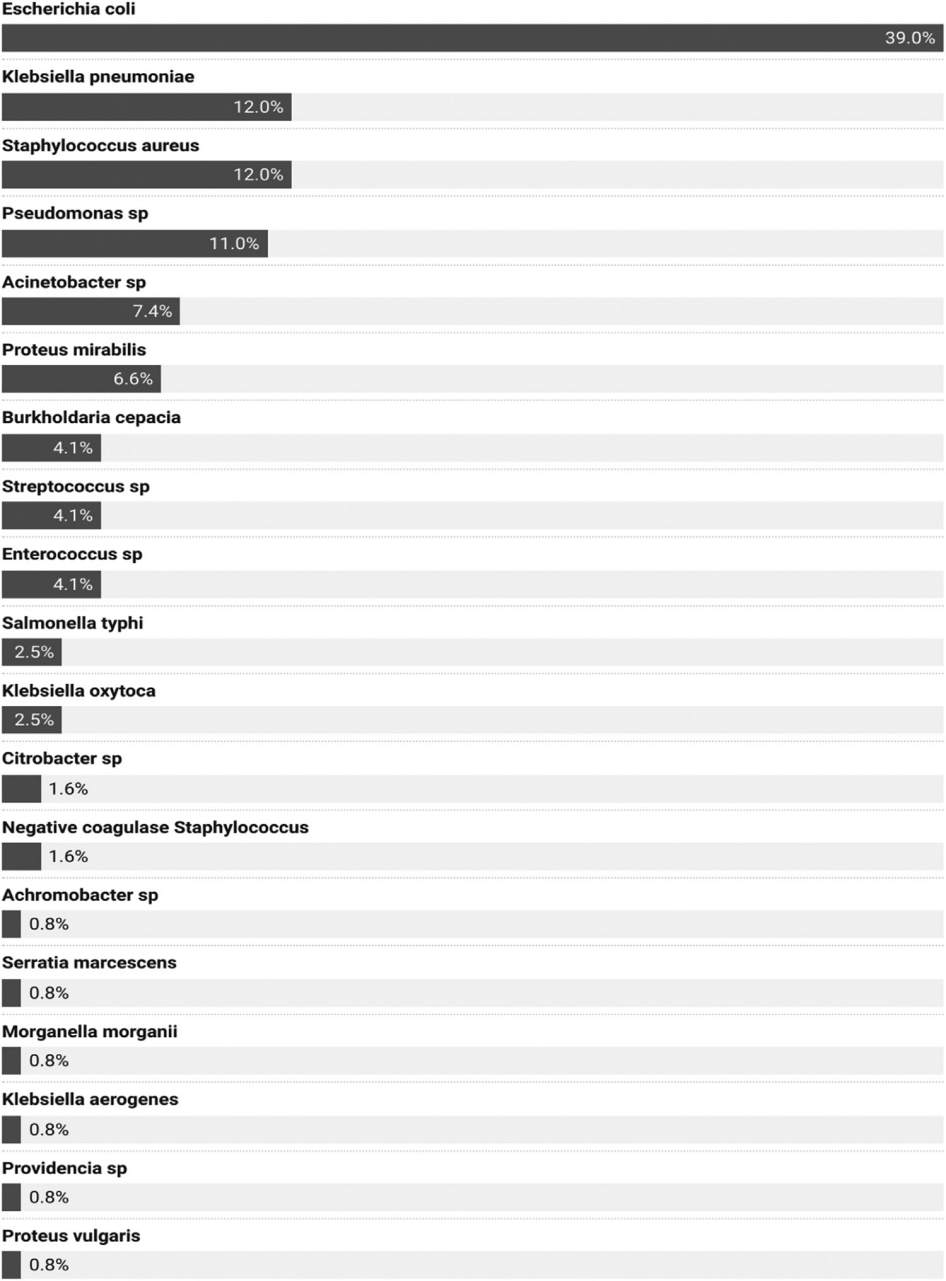
Distribution of bacterial species in patients with bacterial infections in the infectious diseases and internal medicine departments of DPH from January 1, 2023 to December 31, 2023 (N = 123). DPH, Principal Hospital of Dakar.

In this study, bacteria were isolated in 123 patients (68%), with at least one of the 140 bacterial species identified. The most frequently isolated species were *E. coli* (in 48 patients, 39%), followed by *S. aureus* (15 patients, 12%), *K. pneumoniae* (15 patients, 12%), and *Pseudomonas aeruginosa* (14 patients, 11%) (Figure 3).

## Multidrug-resistant bacteria

**Figure 4:**
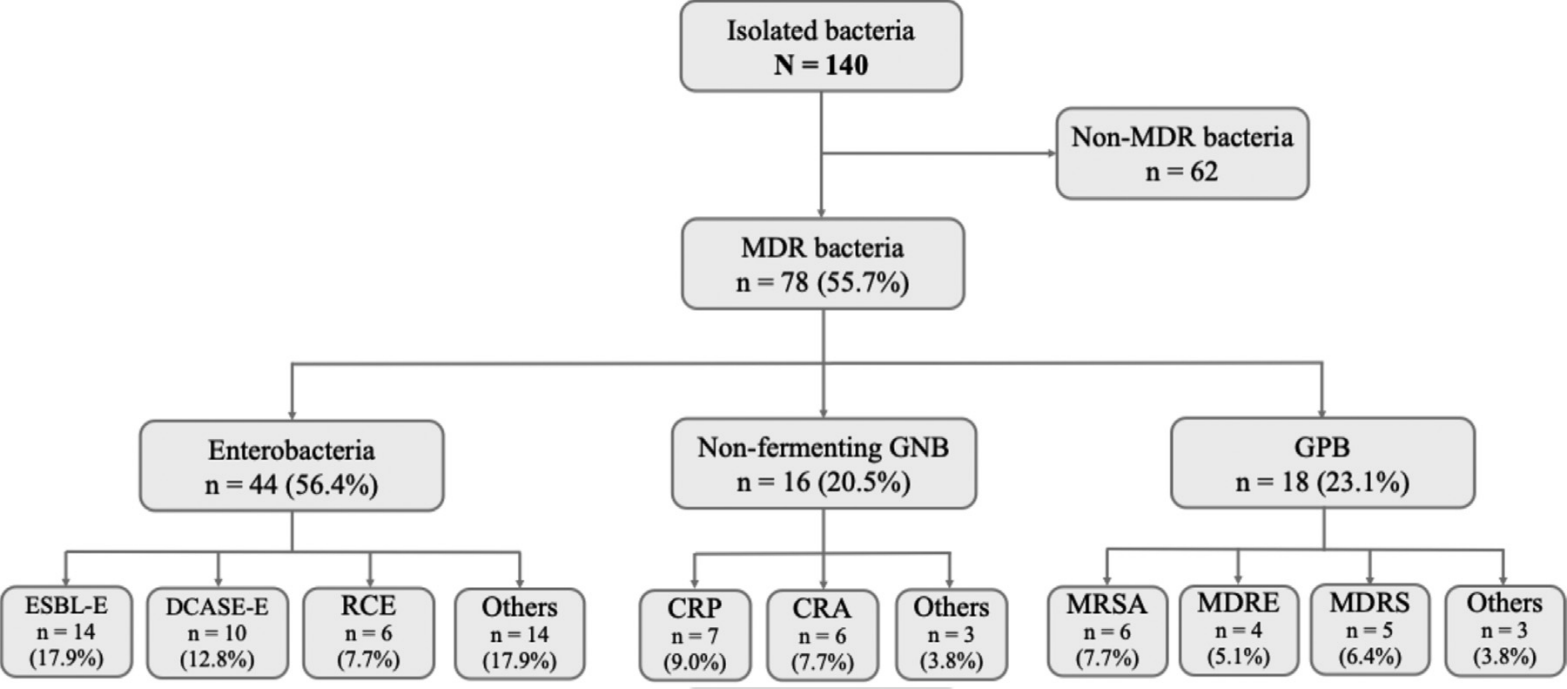
Distribution of MDR bacteria in patients with bacterial infections in the infectious diseases and internal medicine departments of DPH from January 1, 2023 to December 31, 2023 (N = 123). CRA, carbapenem-resistant *Acinetobacter*; CRE, carbapenem-resistant Enterobacteriaceae; CRP, carbapenem-resistant *Pseudomonas*; DCASE-E, derepressed cephalosporinase producing Enterobacteriaceae; DPH, Principal Hospital of Dakar; ESBL-E, extended-spectrum beta-lactamases producing Enterobacteriaceae; GNB, Gram-negative bacteria; GPB, Gram-positive bacteria; MDR, multidrug-resistant; MDRE, multidrug-resistant enterococcus; MDRS, multidrug-resistant streptococcus; MRSA, methicillin-resistant *Staphylococcus aureus*.

Among the 123 patients with confirmed bacterial infections, 64 were infected with at least one MDR bacteria, representing 52%. A total of 78 out of 140 isolated bacteria were MDR, accounting for 55.7%. More than half of these MDR bacteria were enterobacteria, of which 13.6% (6/44) were carbapenem-resistant. Methicillin-resistant *S. aureus* accounted for 33.3% of Gram-positive bacteria (6/18) and 7.7% of all MDR bacteria (6/78) (Figure 4).

### Antibiotic therapy

**Figure 5:**
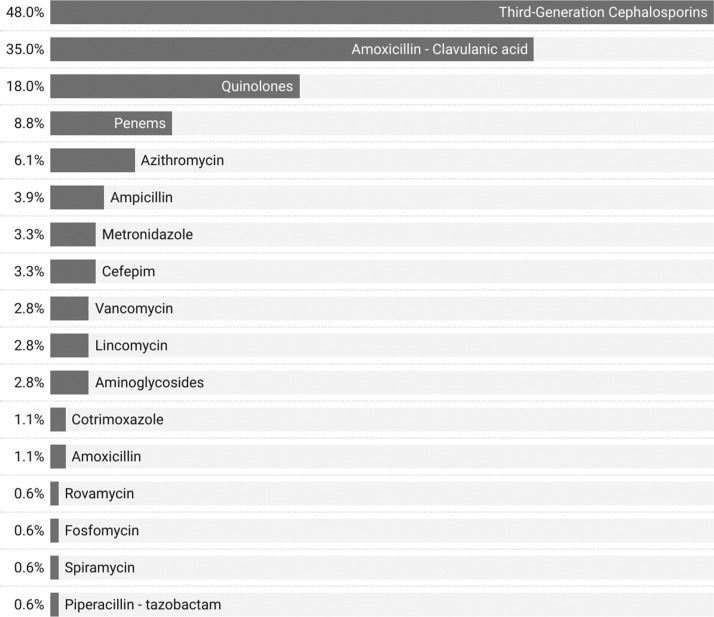
Distribution of antibiotic therapy in patients with bacterial infections in the infectious diseases and internal medicine departments of DPH from January 1, 2023 to December 31, 2023 (N = 175). DPH, Principal Hospital of Dakar

Among the 181 patients, 175 (96.7%) received empiric antibiotic therapy for a mean duration of 6.8±4 days. The adjustment of this empiric antibiotic therapy was made in 45.1% of cases (79/175). The median time to adjustment was 4 days, with extremes ranging from 1 to 33 days. The median total duration of antibiotic therapy was 9 days, with extremes ranging from 1 to 40 days. The most commonly prescribed antibiotics were third-generation cephalosporins (3GC) in 87 patients (48%), followed by amoxicillin-clavulanate in 64 patients (35%), and quinolones in 32 patients (18%) (Figure 5).

### Patients’ outcome

The mean duration of hospitalization was 16±10 days. There were 140 patients (77%) who completely recovered; thus, they were discharged, and 35 patients (19%) who continued antibiotic therapy at home. A total of 28 deaths were recorded, resulting in a death rate of 15%.

### Factors associated with death

Bivariate analysis revealed that advanced age (*P* = 0.042), a history of hospitalization in the last 6 months (*P* = 0.02), the presence of sepsis (*P* = 0.023), low hemoglobin levels (*P* <0.001), and elevated CRP levels (*P* = 0.006) were significantly associated with death (Table 2).

**Table 2:** Bivariate assessment of factors associated with death in patients with bacterial infections in the infectious diseases and internal medicine departments of DPH from January 1, 2023 to December 31, 2023 (N = 181).

Features	Death		p
	Yes (N = 28)	No (N = 153)	
Age (years) [mean±SD]	67±18	59±18	0.042
Sex			>0.9
Female	13 (15%)	73 (85%)	
Male	15 (16%)	80 (84%)	
High blood pressure			0.6
Yes	13 (17%)	62 (83%)	
No	15 (14%)	91 (86%)	
Diabetes			0.7
Yes	13 (17%)	65 (83%)	
No	15 (15%)	88 (85%)	
Kidney disease			>0.9
Yes	1 (10%)	9 (90%)	
No	27 (16%)	144 (84%)	
Heart disease			0.6
Yes	0 (0%)	8 (100%)	
No	28 (16%)	145 (84%)	
Cancer			>0.9
Yes	1 (17%)	5 (83%)	
No	27 (15%)	148 (85%)	
HIV infection			0.7
Yes	2 (18%)	9 (82%)	
No	26 (15%)	144 (85%)	
Previous hospitalization			0.020
Yes	18 (23%)	62 (78%)	
No	10 (9.9%)	91 (90%)	
Respiratory infections			>0.9
Yes	4 (13%)	26 (87%)	
No	23 (16%)	122 (84%)	
Urinary Tract infections			0.9
Yes	9 (15%)	52 (85%)	
No	18 (16%)	96 (84%)	
Digestive infections			>0.9
Yes	3 (14%)	19 (86%)	
No	24 (16%)	129 (84%)	
Bone and join infections			0.6
Yes	0 (0%)	7 (100%)	
No	27 (16%)	141 (84%)	
Neurologic infections			>0.9
Yes	1 (9.1%)	10 (91%)	
No	26 (16%)	138 (84%)	
Skin infections			0.4
Yes	6 (21%)	22 (79%)	
No	21 (14%)	126 (86%)	
Soft tissue infections			0.060
Yes	5 (33%)	10 (67%)	
No	22 (14%)	138 (86%)	
Sepsis (qSOFA ≥2)			0.023
Yes	15 (24%)	48 (76%)	
No	13 (11%)	105 (89%)	
Hemoglobin level (g/dl)			<0.001
≤7	6 (33%)	12 (67%)	
8-11	18 (24%)	58 (76%)	
>12	4 (4.6%)	83 (95%)	
C-reactive protein (mg/l) [median (interquartile range)]	207 (115-304)	110 (58-200)	0.006
Nosocomial infection			0.2
Yes	11 (20%)	43 (80%)	
No	17 (13%)	110 (87%)	

DPH, Principal Hospital of Dakar; qSOFA, quick Sequential Organ Failure Assessment.

**Figure 6:**
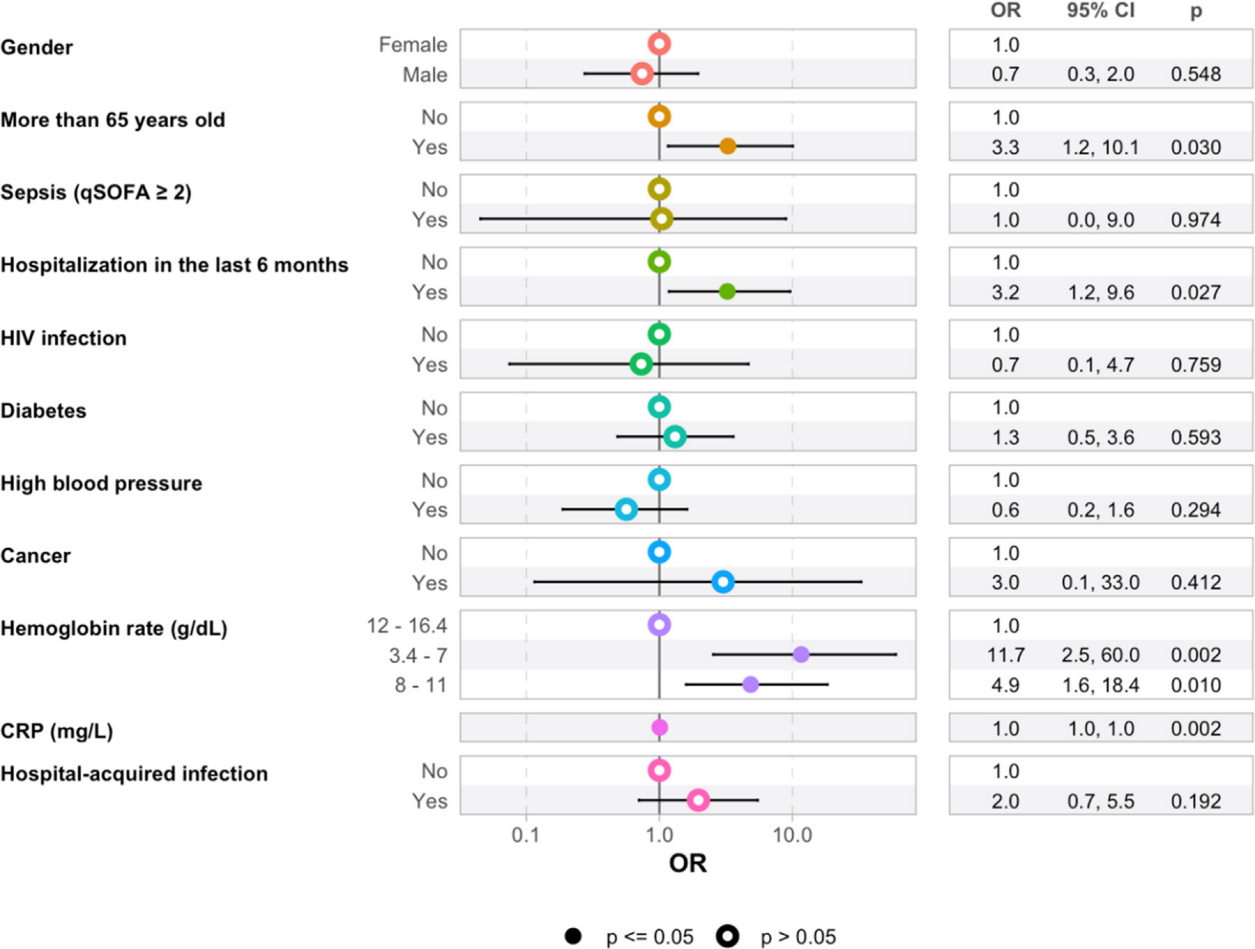
Multivariate assessment of factors associated with death in patients with bacterial infections in the infectious diseases and internal medicine departments of DPH from January 1, 2023 to December 31, 2023 (N = 181). CI, confidence interval; CRP, C-reactive protein; DPH, Principal Hospital of Dakar; qSOFA, quick Sequential Organ Failure Assessment.

After adjusting for potential confounders using multivariate logistic regression, the following factors were associated with mortality: age ≥65 years (OR = 3.2; 95% CI 1.2-9.5, *P* = 0.03), prior hospitalization (OR = 2.9; 95% CI 1.1-8.5, *P* = 0.027), elevated CRP (with a weak association, *P* = 0.002), and low hemoglobin levels. The likelihood of death was nearly 11.7 times higher in patients with hemoglobin levels between 3 and 7 g/dl and 4.9 times higher in those with hemoglobin levels between 7 and 11 g/dl (Figure 6).

## Discussion

### Prevalence of bacterial infection

The prevalence of bacterial infections in this study was 16.7% (22.5% in the infectious diseases department and 13.7% in the internal medicine department). Although we did not find similar studies in Africa for comparison, this hospital prevalence seems relatively high. It is higher than the prevalence found by Melliez et al. [9] among HIV-positive patients, where the prevalence was only 5.5% (1,414/25,795). This discrepancy could be due to the lower socioeconomic level in Senegal, which favors the more frequent occurrence of infectious diseases, mainly bacterial infections.

### Etiologies

The most frequently identified etiologies in our study were *E. coli* (39%), followed by *S. aureus* (12%), *K. pneumoniae* (12%), and *P. aeruginosa* (11%). In the majority of studies, these four pathogens are responsible for bacterial infections. This was the case in the study by Wembulua et al. [10] in Dakar, Diedhiou et al*.* [11] in Saint Louis (Senegal), Rahden et al. [12] in The Gambia, and Kakupa et al. [13] in the Democratic Republic of Congo. A recent systematic review has shown that these four bacteria are among the five most frequent pathogens responsible for severe infections and 54.9% of deaths among the 33 bacteria studied [1]. Indeed, these four pathogens have high environmental adaptability, virulence, and protective factors such as pili, toxins, capsules, or enzymes, and they are also able to exploit certain contexts like immunosuppression or invasive procedures and increasing resistance to antibiotics.

However, in 58 patients (32%), the causative bacteria were not identified. This is quite common in African studies due to non-compliance with sample collection and transport conditions, limited laboratory facilities, and early administration of antibiotics before samples are collected, which makes bacteria identification difficult. Moreover, in certain infections, such as acute community-acquired pneumonia and skin infections, the request for microbiologic tests to document the infection is not always necessary.

### Multidrug-resistant bacteria

Among the 140 bacteria identified and tested for antibiotic susceptibility, 78 (55.7%) were MDR and were isolated in 52% of patients. Higher or similar MDR prevalence rates have also been reported in many other sub-Saharan African countries. In the studies by Bonko et al. [14] in Burkina Faso and Ombelet et al. [15] in Benin, MDR bacteria represented 54.6% and 73.9% of isolated bacteria, respectively. A systematic review conducted in Ethiopia, including 37 studies, estimated the prevalence of MDR bacteria at 70.5% [16]. MDR bacteria appear to be less prevalent in Maghreb countries and in developed countries. Indeed, MDR prevalence rates of 26.4% and 14% were reported in Tunisia [17] and Morocco [18], respectively. Similarly, in patients hospitalized for cirrhosis in hospitals across northern, southern, and western Europe, the prevalence of MDR bacteria was 29.2% [19]. These findings support the increasing trend of MDR infections in sub-Saharan African countries over the years. This increase could be attributed to several factors, including the misuse of antibiotics favored by poor antimicrobial management policies, inadequate pathogen surveillance across the humans, animals, and environmental sectors, as well as the insufficient implementation of hygiene measures.

### Factors associated with death

The death rate found in our study was high, representing 15%. Several factors associated with these deaths were identified through multivariate analysis. Patients aged ≥65 years were 3.2 times more likely to die than those younger than 65 years old. This association is reported in nearly all prognostic studies on bacterial infections available in the literature. For instance, in a large multicenter cohort in the United States collected over a 24-year period (1979-2002) [20], mortality related to sepsis was 2.26 times more likely in patients aged ≥65 years. In Algeria, Dali-Ali et al*.* [21] also found a statistically significant link (*P* = 0.01) between advanced age and mortality in their case-control study on patients with nosocomial infections in intensive care settings. This association is explained by the immune deficiencies associated with aging and the frequent presence of underlying comorbidities in this group, which make them more vulnerable to bacterial infections.

The history of hospitalization within the last 6 months preceding inclusion was also associated with the occurrence of death in the patients included (OR = 2.9; 95% CI 1.1-8.5). Previously hospitalized patients may carry severe pathologies justifying their readmission. They also have an increased risk of being infected with MDR bacteria of nosocomial origin, which worsens their prognosis.

In this study, the lower the hemoglobin level, the higher the probability of death. Indeed, compared with patients with hemoglobin levels >11 g/dl, those with levels between 3 g/dl and 7 g/dl and between 8 g/dl and 11 g/dl were 11.7 and 4.9 times more likely to die, respectively. In the findings of Oh et al*.* [22] in South Korea, patients with bacterial infections and anemia were 1.77 times more likely to die (*P* <0.001). The same observation was made by Amrani et al. [23] in a children's hospital in Rabat (Morocco) in patients admitted for bacterial meningitis (OR = 8.43, 95% CI 1.57-45.26). Anemia, particularly when severe, can lead to reduced oxygen transport, resulting in tissue hypoxia and, consequently, multi-visceral suffering. Furthermore, low hemoglobin levels weaken the immune system, making the body less able to fight invasive bacterial infections.

As for positive CRP, it was significantly associated with death in multivariate analysis (*P* = 0.002), but this link was weak (OR = 1). An association between CRP and bacterial infection-related deaths was reported by Burlaud et al. [24] in France in older adult patients with bacteremia (*P* = 0.02). Djuma et al. [25] also demonstrated that high CRP levels were closely related to mortality in patients admitted to intensive care. Elevated CRP is often correlated with an intense systemic inflammatory response, which often indicates a complication of the bacterial infection, such as sepsis or septic shock. Excessive CRP elevation can also lead to multi-visceral dysfunction, a key factor in vital prognosis.

### Limitations of the study

Although our study provides valuable insights, some of its limitations are to be pointed. First, the relatively small sample size may affect the power of the statistical analyses. Furthermore, the monocentric nature of this study prevents statistical inference from the results obtained. Limited laboratory facilities in the context of a resource-constrained country restricted our ability to identify certain bacteria as well as their resistance phenotypes and genotypes. Finally, during the assessment of factors associated with death, some potentially explanatory factors, such as socioeconomic status and the delay in diagnosis and management, could not be collected.

## Conclusion

This study highlights the significant morbidity and mortality associated with bacterial infections in the infectious diseases and internal medicine departments, with a notable prevalence of MDR bacteria. It also identified several factors associated with mortality from these infections, such as advanced age, prior hospitalization, and the severity of anemia. Considering these prognostic factors in the management of patients with bacterial infections could help improve survival outcomes. Furthermore, strengthening standards and additional hygiene measures contribute to the prevention of these infections.

## Declarations of competing interest

The authors have no competing interests to declare.

## Appendix Supplementary materials

Image, application 1
