# Supplementary material for: Prevalence of bacterial infections and factors associated with death related to these infections in two medical departments of a tertiary hospital in Dakar, Senegal
Source: IJID Reg. 2025 Mar 8;15:100623. doi: 10.1016/j.ijregi.2025.100623 (PMC11994947; doi:10.1016/j.ijregi.2025.100623)
Supplement: Supplementary file 1 [file mmc1.pdf]

Docusign Envelope ID: B7441283-B2CA-4168-8573-FDAE66A8E1B7

REPUBLIQUE DU SENEGAL

MINISTERE DE LA SANTE ET DE L'ACTION SOCIALE

HOPITAL GENERAL IDRISSA POUYE

COMITE ETHIQUE

Dakar le 11 septembre 2024

Au Dr Moustapha DIOP
Service Maladies infectieuses,
Santé publique
Hôpital Principal Dakar
SENEGAL

Référence : Projet Recherche 003/2024/CETH/HOGIP : «Prévalence des infections bactériennes non spécifiques et facteurs associés au décès lié à ces infections aux services de médecine interne et des maladies infectieuses de l'Hôpital Principal de Dakar du 1er janvier 2023 au 31 décembre 2023 »

APPROBATION ETHIQUE DE LA RECHERCHE

Votre protocole de recherche a été examiné selon les règles édictées par le Comité Ethique (CETH) de l'Hôpital Général Idrissa Pouye (HOGIP) et conformément aux procédures établies par ledit comité pour l'approbation éthique de toute recherche impliquant des participants humains.

Nous avons le plaisir de vous informer que, sur la base des informations fournies par votre équipe, le Comité Ethique (CETH) de l'HOGIP considère que la recherche proposée, respecte les normes éthiques appropriées et en conséquence, approuve son exécution.

Le CETH attire votre attention sur tout changement ultérieur dans la recherche qui soulèverait des questions éthiques non incluses dans le protocole original. Ces changements devront être soumis au Comité Ethique pour approbation.

DS

9/11/2024

Le Président

DocuSigned by:

Pr Mohamed M. SOUMAH

Mohamed M. Soumah

3437BD90C7114CD...
